# Quantitative analysis of CT-perfusion parameters in the evaluation of brain gliomas and metastases

**DOI:** 10.1186/1756-9966-28-38

**Published:** 2009-03-16

**Authors:** Anna Maria Di Nallo, Antonello Vidiri, Simona Marzi, Alessandra Mirri, Alessandra Fabi, Carmine Maria Carapella, Andrea Pace, Marcello Crecco

**Affiliations:** 1 Regina Elena National Cancer Institute, Rome, Italy; 2 Ospedale San Filippo Neri, Rome, Italy

## Abstract

**Background:**

The paper reports a quantitative analysis of the perfusion maps of 22 patients, affected by gliomas or by metastasis, with the aim of characterizing the malignant tissue with respect to the normal tissue. The gold standard was obtained by histological exam or nuclear medicine techniques. The perfusion scan provided 11 parametric maps, including Cerebral Blood Volume (CBV), Cerebral Blood Flow (CBF), Average Perfusion (P_mean_) and Permeability-surface area product (PS).

**Methods:**

The perfusion scans were performed after the injection of 40 ml of non-ionic contrast agent, at an injection rate of 8 ml/s, and a 40 s cine scan with 1 s interval was acquired. An expert radiologist outlined the region of interest (ROI) on the unenhanced CT scan, by using a home-made routine. The mean values with their standard deviations inside the outlined ROIs and the contralateral ROIs were calculated on each map. Statistical analyses were used to investigate significant differences between diseased and normal regions. Receiving Operating Characteristic (ROC) curves were also generated.

**Results:**

Tumors are characterized by higher values of all the perfusion parameters, but after the statistical analysis, only the *PS*, *Pat*_*Rsq *_(Patlak Rsquare) and *T*_*peak *_(Time to Peak) resulted significant. ROC curves, confirmed both *Pat*_*Rsq *_and *PS *as equally reliable metrics for discriminating between malignant and normal tissues, with areas under curves (AUCs) of 0.82 and 0.81, respectively.

**Conclusion:**

CT perfusion is a useful and non invasive technique for evaluating brain neoplasms. Malignant and normal tissues can be accurately differentiated using perfusion map, with the aim of performing tumor diagnosis and grading, and follow-up analysis.

## Background

CT perfusion is a technique that provides information on brain hemodynamics by analyzing the first passage into the cerebral vessels of an intravenous contrast bolus. The technique can be rapidly performed using a multislice CT scanner, capable of producing the cine mode scan with the aid of an automatic injector; the perfusion maps are generated by a workstation running dedicated software.

Modern imaging systems, being completely digital, are suitable for quantitative analyses [1-3].

In particular, CT-Perfusion imaging permits a qualitative and quantitative evaluation of the brain perfusion by mapping cerebral blood flow (CBF) and cerebral blood volume (CBV).

The Perfusion-CT technique has been found to be useful in the evaluation of cerebral ischemia and infarction, but recent studies have investigated the role of perfusion maps for evaluating brain neoplasms, because there is growing interest in the non-invasive assessment of tumor vascularity [4]. The rationale for the use of CT Perfusion for neoplasms is that the technique provides information about tumor angiogenesis. The increase of angiogenic activity and neovascularization in the neoplasms results in an increase of microvascular permeability and CBV, related to the presence of immature, disrupted or absent vessels of the blood-brain-barrier (BBB).

In recent studies [5-9], CT-Perfusion imaging of brain tumors has been shown to be helpful for assessing preoperative tumor grade, differentiating between the tumor enhancement and the radiation necrosis; evaluating the response to anti-angiogenetic agents as well as guiding biopsy procedure, when the biopsy target is chosen on the basis of the identification of the hypervascularization area inside heterogeneous tumors.

The aim of this study was to use perfusion maps to characterize malignant versus normal tissue, in order to select those parameters to be used in subsequent clinical studies for a more accurate diagnosis.

## Methods

### Patients

A 4 slices helical CT scanner (Somatom Plus 4 Volume Zoom; Siemens Medical Systems, Erlangen, Germany) was used and perfusion CT was incorporated into the patients' conventional CT examination. The study was approved by our institutional review board and informed consent was obtained from all patients.

A total of 22 patients were enrolled in this study: 12 patients affected by malignant gliomas (7 Glioblastoma (GBM), 2 by Anaplastic Astrocytoma (AA), 2 by Oligodendrogliomas), 10 patients affected by metastases (from 6 breast, 2 lung, and one melanoma and maxillary sinus cancers). The patient's clinical and histological information is reported in Table 1.

**Table 1:** Clinical and histological information of the group of 22 patients included in the study

Patient no.	Sex	Pathology	Patient status
1	M	Melanoma	Recurrence
2	F	Breast Cancer	Recurrence
3	F	Breast Cancer	Recurrence
4	F	Breast Cancer	Recurrence
5	M	Maxillary sinus	Metastasis
6	F	Breast Cancer	Metastasis
7	F	Breast Cancer	Metastasis
8	M	Glioblastoma	Recurrence
9	M	Astrocytoma	Recurrence
10	M	Oligodendroglioma	Recurrence
11	M	Glioblastoma	Recurrence
12	M	Glioblastoma	Recurrence
13	M	Glioblastoma	Recurrence
14	M	Glioblastoma	Recurrence
15	M	Glioblastoma	Recurrence
16	M	Glioblastoma	Recurrence
17	M	Lung Cancer	Recurrence
18	M	Astrocytoma	Recurrence
19	M	Breast	Metastasis
20	M	Oligodendroglioma	Recurrence
21	M	Lung	Metastasis
22	F	Lung	Metastasis

CT-Perfusion was performed in 11 patients for the differential diagnosis between a tumor relapse or a radiation necrosis on the basis of morphologic imaging (MR, Magnetic Resonance); in 8 patients during post radiation and/or chemotherapy follow-up; in 3 patients, before diagnosis, to evaluate the area of maximum vascularization at the site of the stereotactic biopsy.

The diagnostic status of the patients should be known with certainty (Gold Standard). Depending on the clinical task, histopathological exam, follow-up of the lesion, diagnosis by a panel of experts or information about cause-of-death could all be useful to define the gold standard. In particular, the length of the follow-up study is based on reasonable estimates of cancer growth rates.

Because the present study is retrospective, the method used to determine the patient status depends on a single case. All the primary tumors were detected through histological diagnosis, surgical resection or stereotactic biopsy. In some cases, where the diagnosis was undefined, the gold standard was obtained by nuclear medicine techniques: SPECT (Single Photon Emission Computed Tomography) in 4 patients and PET-CT (Positron Emission Tomography) with FdG (fluoro-deoxy-glucose) or Methionine in 4 other patients. In some cases, particularly for patients affected by metastasis, who underwent surgery for the differential diagnosis between tumor relapse or radiation necrosis, the gold standard was histological data and for the patients who did not undergo surgery a three or six month follow-up, showing lesion regression or tumor progression, was considered.

All the lesions included in this study, both primary and secondary, were investigated by morphological MR, utilizing a superconductive magnet, operating at 0.5 T; SE (spin-eco) technique and T1, T2-weighted and FLAIR (Fluid Attenuated Inversion Recovery) sequences were used before contrast medium infusion, after injection of a double-dose of Gadolinium-DTPA (diethylenetriamine penta-acetic acid), SE T1 sequences in axial, coronal and sagittal planes were used.

### CT perfusion technique

After un-enhanced CT of the whole brain to detect the lesion, two adjacent 10 mm. thick sections were selected in the area of interest, at level of the largest transverse lesion dimension. The perfusion scan was performed after the injection of 40 ml of non-ionic contrast agent containing 300 mg of iodine per ml (Iopamidol or Omnipaque; Nycomed, Oslo, Norway), at an injection rate of 8 ml/s; the time of total infusion by the automatic injector was 5 s. Four seconds after the injection began, a 40 s cine (continuous) scan with 1 s interval was acquired at the chosen slice location.

The patients received the total effective dose equivalent to 1.1 mSv according to other values in the literature [10-12] calculated by ImPACT CT Patient Dosimetry Calculator (v. 0.99×, Medical Devices Agency, London).

### Image analysis

CT perfusion data (40 grow images in a gray scale) were analyzed and elaborated by a Siemens imaging workstation equipped with CT Perfusion; Access SOMATOM Sensation 4 software (Version A47C) to yield coloured map images.

The Anterior Cerebral Artery (ACA) or middle cerebral artery (MCA) was selected as input artery, and a large venous structure, such as the torcular herophili is chosen as the input vein. Particular attention was given to the selection of the arterial and venous input functions and to the choice of the cut-off values for unenhanced and enhanced images. To avoid partial volume effects a reference vessel large enough and sufficiently orthogonal to the scan section was selected.

The elaborated images are represented by 11 parametric maps: a standard set including the Maximum Intensity Projection (MIP), Cerebral Blood Volume (CBV), Cerebral Blood Flow (CBF) and Time to Peak (T_peak_) maps and an optional set including the Average Perfusion (P_mean_), Peak Enhancement (Peak_Enh_), Time to Start (T_start_), Permeability (PS = permeability-surface area product), Patlak Rsquare (Pat_Rsq_), Patlak Residual Perfusion (Pat_Res_)and Patlak Blood Volume (PBV).

The Peak Enhancement, Time to Start and to Peak Perfusion, Average Perfusion are semi-quantitative parameters, readily obtained from the tumor attenuation curve that reflects the tumor vascularity.

It is known that the perfusion can be calculated either from the maximal slope of the tissue concentration-time curve or from its peak height, normalized to the arterial input function [13]. The modelling used by the commercial software is based on compartmental analysis: a two compartment model (intravascular equivalent to blood and extravascular equivalent to tissue extracellular fluid) is used by assuming the back flux of contrast medium from extravascular to intravascular compartments to be negligible for the first 1 to 2 min (a technique known as Patlak analysis [14]).

On the basis of this theoretical model, the exchange between the blood and the tissue can be well described by the Patlak plot, representing the ratio of tissue to blood concentration against the ratio of the AUC (area under curve) of the blood curve to the blood concentration for various time values. If the data are consistent with this theoretical model then the plot is linear (Pat_Rsq _R^2 ^→ 1 e Pat_Res _σ^2 ^→ 0), with a slope equal to the blood clearance per unit volume (Permeability) and an intercept equal to the tissue's relative blood volume (PBV).

**Figure 1:**
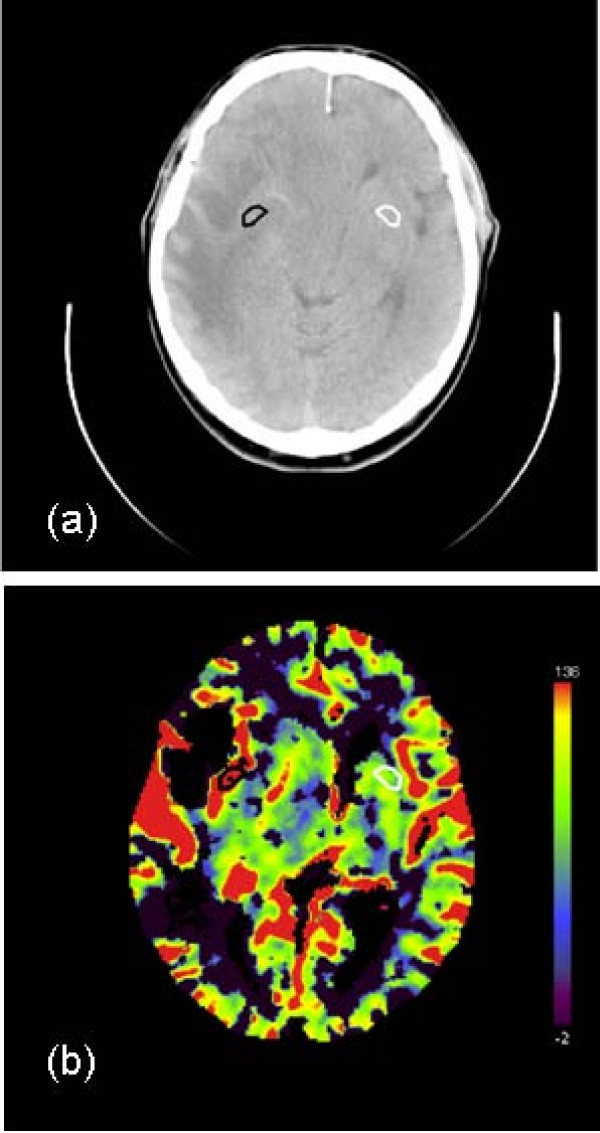
**Transverse CT (Computer Tomography) image (a) and CBV (Cerebral Blood Volume) map (b) in a patient with grade III astrocytoma**. In both the images, the hand-drawn ROI (region of interest) corresponding to the tumor and the contralateral ROI are displayed in black and white, respectively.

**Figure 2:**
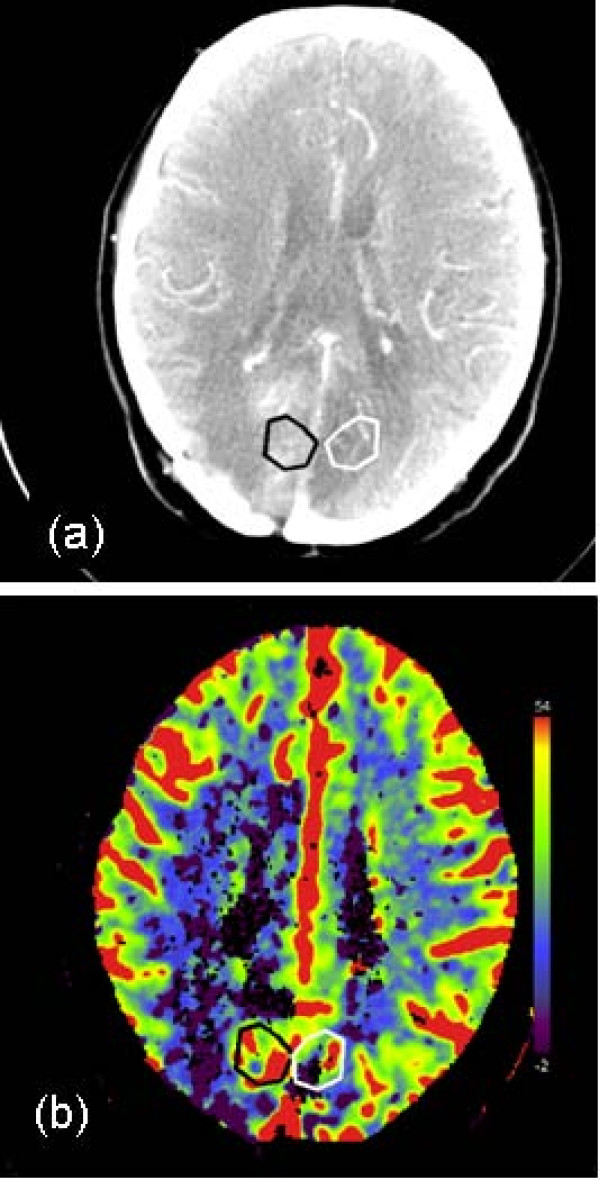
**Transverse CT (Computer Tomography) image (a) and CBV (Cerebral Blood Volume) map (b) in a patient affected by a recurrence of glioblastoma**. In both the images, the hand-drawn ROI (region of interest) corresponding to the tumor and the contralateral ROI are displayed in black and white, respectively.

Both the elaborated and row images were exported by means of the Digital Imaging and Communications in Medicine (DICOM) protocol to a personal computer for a post-processing procedure. This consists of a manual selection of a ROI by an expert radiologist on the unenhanced CT scan, according to the alternative functional imaging exams (MR or PET). In Fig. 1 the transverse CT image and the CBV map in a patient with grade III astrocytoma, are shown; ROIs representing the lesion and the contralateral region are displayed in black and white respectively. To underline the variability in volume size of tumors, another example of a patient, affected by a recurrence of glioblastoma, is shown in Fig. 2.

### Quantitative analysis

Being completely digital, the images were suitable for quantitative analyses, pixel per pixel. Home-made software has been developed using Matlab code (Release 6.5, The Mathworks Inc., Natick, Massachusetts) to perform quantitative analyses. This software permits the parametric maps obtained by CT perfusion data sets to be visualized, displaying the data type (CBV or CBF etc.), the slice position and the file name on each map. A graphic tool was developed to allow the radiologist to place an arbitrary ROI on each image, obtaining the corresponding area size and the mean value with its standard deviation inside the drawn ROI. The side-to-side ratios of these values have been automatically calculated from mirrored regions in the contralateral hemisphere. Particular attention was paid to exclude that the automated contour of the contralateral region included arterial or venous structures, altering data and affecting the subsequent statistical analyses.

All elaborated data, corresponding to the mean values with their standard deviations inside the outlined ROIs, the contralateral ROIs and their ratios were recorded in an output text file. These data were initially used to investigate whether some perfusion parameters coming from CT perfusion data could be useful to characterize the entire patient group. Later, the diseased region (malignant glioma or metastases), and the contralateral region (normal tissue) were studied to find out if they could be differentiated on the basis of some parametric maps. The more significant parameters for differentiating between lesion and normal tissue were obtained through a statistical analysis.

### Statistical analysis

ROC analysis [15] was used to compare the accuracy of the radiological tests in identifying and discriminating diseased from normal cases in a five-point scale classification (normal, benign, probably benign, probably malignant and malignant. A ROC curve for these five decision thresholds corresponding to the number of true positive, true negative, false positive and false negative cases was plotted.

In a ROC curve the Sensitivity (TPF: True Positive Fraction) is plotted in function of the (1-Specificity) or (FPF: False Positive Fraction), where Specificity is (TNF: True Negative Fraction).

Only all the values of sensitivity/specificity pairs plotted in the roc curve provides a complete picture of test accuracy and the area under the ROC curve (A_z_) is the measure [16].

A computer software packages NCSS (Release NCSS2007, Kaysville, Utah) was used to determine the statistical significance (p-values) of the difference between the areas under ROC curves with the relative standard error and 95% confidence interval.

In addition to ROC curves, parametric (t-test for independent and paired samples) and non-parametric tests (Wilcoxon Signed Ranks test) were also used to investigate the statistically significant differences between diseased and normal regions.

## Results

**Figure 3:**
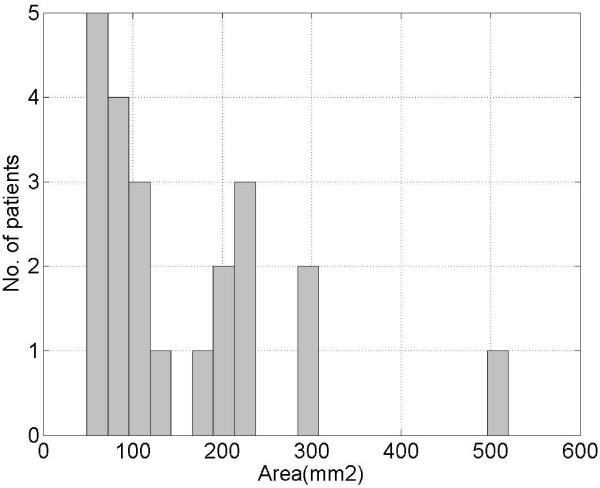
**Histogram of the areas outlined by the radiologist, for each patient, as malignant regions**.

Before evaluating the parametric maps, an analysis of the tumor size was made for the patient population included in this study. In Fig. 3 the histogram of the areas outlined by the radiologist for each patient as malignant region has been displayed. The average area being 157.0 mm^2 ^and the range was 48.6–520.0 mm^2^. This analysis was performed on the evidence that the great variability in ROI size surely has a great impact on the mean perfusional values and their variability inside ROIs (see also Fig. 1, 2).

Using perfusion maps to find the possible predictors of malignancy, an analysis was performed on 22 patients affected by a malignant glioma or metastases. The mean values and the standard deviations of all the parameters inside the ROIs delineated by the radiologist as lesions and inside the contralateral ROIs were calculated and shown in Table 2 (T_start _was not included being considered of minor interest for the aim of the study).

**Table 2:** Average values and standard deviations of all the perfusional parameters for malignant and normal tissue.

	***Pat*_*Res*_*(1:1000)***	***Pat*_*Rsq*_*(1:100)***	***PS(0.5 ml/100 ml/min)***	***PBV(%)***	***T*_*peak*_*(s)***
***Normal Tissue***	9.0 ± 5.7	8.5 ± 9.0	4.2 ± 6.9	3.3 ± 1.6	5.4 ± 2.2
***Lesion***	10 ± 5.3	34.6 ± 29.3	14.2 ± 12.6	4.0 ± 1.8	7.5 ± 2.7

					

	***CBV(%)***	***Peak*_*enh*_*(a.u.)***	***CBF(ml/100 ml/min)***	***P*_*mean*_*(a.u.)***	***MIP(a.u.)***

***Normal Tissue***	4.3 ± 3.2	7.8 ± 8.3	30.9 ± 24.7	35.8 ± 15.0	50.0 ± 16.2
***Lesion***	6.3 ± 5.0	10.9 ± 8.0	38.8 ± 40.0	42.9 ± 15.0	55.7 ± 12.5

The relative units are indicated in brackets (a.u. is an abbreviation for arbitrary units).

Both parametric (t-test) and non-parametric tests (Wilcoxon Signed Ranks test) were used to perform the study, and the t-test was executed with the hypothesis of both independent and paired samples to exclude the possibility that the values obtained inside the contralateral ROIs could be affected by the presence of a tumor on the other hemisphere (Tab. 3). The non-parametric Wilcoxon Signed Ranks test was performed, as, due to the limited number of patients it could not be established with certainty whether the probability distribution underlying data was normal, results are displayed in Table 4.

**Table 3:** Results of paired samples t-tests, performed to compare the average perfusional values inside the lesion with those inside the contralateral region.

	Paired Samples t-test				
	***Pat*_*Res*_**	***Pat*_*Rsq*_**	***PS***	***PBV***	***T*_*peak*_**

***t***	1.599	3.851	4.161	1.931	2.103
***P***	0.132	**0.002**	**0.000**	0.068	0.054

	***CBV***	***Peak*_*enh*_**	***CBF***	***P*_*mean*_**	***MIP***

***t***	1.727	1.008	0.912	1.443	1.391
***P***	0.106	0.331	0.376	0.171	0.179

P-values < 0.05 are evidenced in bold.

**Table 4:** Results of Wilcoxon Signed Ranks Test, performed to compare the average perfusional values inside the lesion with those inside the contralateral region.

	***Pat*_*Res*_**	***Pat*_*Rsq*_**	***PS***	***PBV***	***T*_*peak*_**
***T***	-1.526	-3.234	-3.564	-1.625	-1.853
***P***	0.127	**0.001**	**0.000**	0.104	0.064

	***CBV***	***Peak*_*enh*_**	***CBF***	***P*_*mean*_**	***MIP***

***T***	-1.563	-1.415	-0.750	-0.909	-0.974
***P***	0.118	0.157	0.453	0.363	0.330

P-values < 0.05 are evidenced in bold.

The ROC curves of parameters found to be statistically significant, ***Pat*_*Rsq*_**, ***PS ***and ***T*_*peak *_**(actually this parameter gave a p slightly superior to 0.05) have also been generated. In Table 5 ROC curve areas and 95% confidence intervals for Pat_Rsq_, PS and T_peak _were reported. By means of the univariate z-score test, it was determined the statistical significance (p_z _value = 0.05) of the difference between each ROC curve and the reference line (diagonal) with area equal to 0.5. The z-test results were also shown in Table 5.

**Table 5:** Areas under the Receiving Operating Characteristic curves (A_z_), 95% confidence intervals and results of the Z test for Pat_Rsq _(Patlak Rsquare), PS (Permeability-surface area product) and T_peak _(Time to Peak).

	**A_z _± SE**	**95% Confidence interval**	**p_z_**
***Pat*_*Rsq*_**	**0. 82 ± 0.08**	0.58 ÷ 0.93	**0.02**
***PS***	**0.81 ± 0.09**	0.57 ÷ 0.92	**0.02**
***T*_*peak*_**	0.68 ± 0.10	0.44 ÷ 0.83	0.12

P-values < 0.05 and the related A_z _are evidenced in bold.

**Figure 4:**
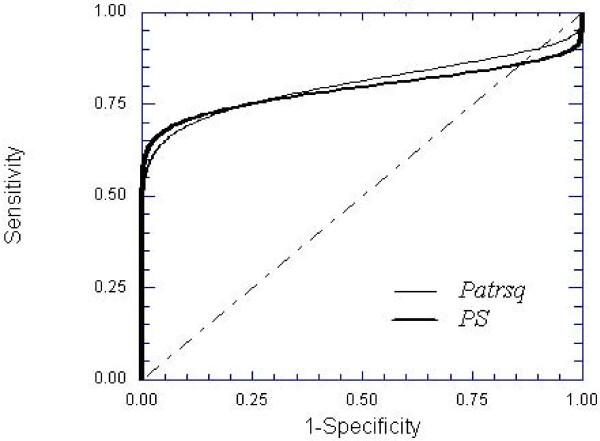
**Receiving Operating Characteristic (ROC) curves of parameters were significant for predicting the presence of malignant tissue (the diagonal represents the reference line with area equal to 0.5)**.

Only the ROC curves of ***Pat*_*Rsq *_**and ***PS***, found to have a significant predictive value for differentiating between malignant and normal tissue were displayed in Fig. 4. The curves are based on the binormal assumption, that was previously verified performing the Kolmogorov-Smirnov normality test. No statistical significant difference was found between the two ROC curves.

To investigate the relationships between the variables found to be significantly correlated with malignancy, a Spearman correlation study was performed (Table 6). Only ***Pat*_*Rsq *_**and ***PS ***resulted correlated, the *R *coefficient being equal to 0.876.

**Table 6:** Spearman's correlation coefficients R and p-values to measure how Pat_Rsq _(Patlak Rsquare), PS (Permeability-surface area product) and T_peak _(Time to Peak) are related.

		**PS**	**T_peak_**
***Pat*_*Rsq*_**	*R*	0.876	0.257
	*p*	**0.000**	0.178

P-values < 0.05 are evidenced in bold.

**Figure 5:**
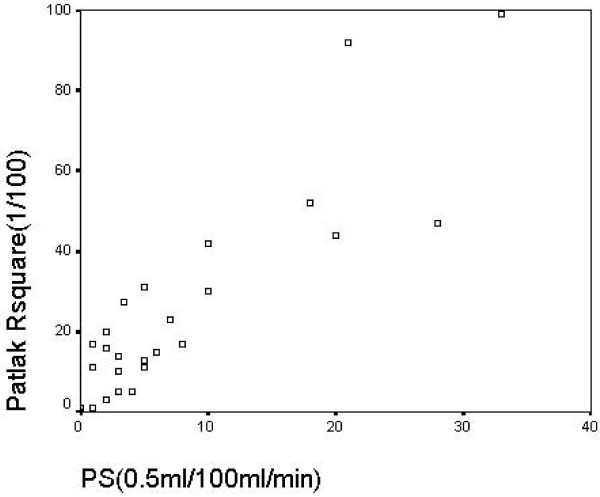
**Plot of *Pat*_*Rsq *_(Patlak Rsquare) versus *PS *(Permeability-surface area product), showing the strong correlation between variables, as confirmed by the Spearman's correlation coefficient equal to 0.876**.

A plot of ***Pat*_*Rsq *_**versus ***PS ***is displayed in Fig. 5, showing a strong correlation between these variables.

## Discussion

Dynamic perfusion imaging with CT or MR is based on the imaging evaluation of biodistribution of the contrast medium infusion acting as a tracer. The contrast medium after infusion is distributed into the tissue in relation to local microvascularization and on the diffusion across the endothelial membrane into the interstitial space. The imaging depicts the distribution of the contrast medium by measuring variations in the vessels and in the tissue enhancement over time. Tissue is composed of three compartments: vascular (capillaries), interstitial and intracellular compartments; the contrast medium used in clinical practice has interstitial diffusion; the interstitial compartment represent the volume into which the contrast diffuses while this contrast does not penetrate the cells or blood cells.

In this study, CT-Perfusion imaging of brain tumors was used to characterize brain tumors and metastases, analyzing the perfusional maps of 22 patients affected by a malignant glioma or metastasis. Always the same radiologist (A.V.) outlined the ROIs identifying the tumor, to reduce the inter-observer variability. In fact, it has been assessed by other authors [17] that the variability in mean quantitative values of CBF, CBV and MTT was less than 9%, among a group of 6 observers with varying levels of skill.

It turned out that tumors are characterized by higher values of all the perfusion parameters, including ***CBV ***and ***CBF***, but, after both parametric and non-parametric statistical tests, only the ***PS***, ***Pat*_*Rsq *_**and ***T*_*peak *_**resulted relevant to identify a neoplastic tissue. In particular, the ***PS***, ***Pat*_*Rsq *_**and ***T*_*peak *_**were on average 3.4, 4 and 1.4 higher for the tumor than for normal tissue, respectively (Table 2). From the high standard deviations of all the parameters it can be inferred that a great variability exists among patients, both inside normal and malignant tissues, as evidenced by other authors [10].

The increased vascular proliferation of the tumor and the hypothesis that feeding arterioles in neoplastic tissue are more vasodilated than in normal tissue are largely supported by previous studies [7-9] and can also explain our findings.

Because of the short scan duration (45 s), the perfusion and blood volume represent the more accurate maps; in fact a study of vascular permeability should have required a scan time up to 2 to 10 min, as suggested by Miles *et al*. [18]. Nevertheless the parameter PS resulted the most sensitive to tissue changes from a normal to malignant state, even if acquired for a partial time. Anyway, several studies reported measurements of vascular permeability using CT scan duration only slightly longer than that one used in the present work [7,19,20].

Probably due to the high standard deviation of our data, reflecting the high heterogeneity of tumor, some parameters did not show the expected predictive power. In fact, in the majority of cases, the region outlined by the radiologist as malignant appears spatially inhomogeneous, with areas of vascular proliferation and areas of necrosis. The presence of necrotic tissue inside the lesion, in particularly in high-grade gliomas and large metastases, surely affects data, decreasing the average values of blood volume, flow and permeability. It can be supposed that, for these reasons, some parameters such as CBV and CBF did not appear to be significant for identifying the lesion, contrary to the results of other authors [7-9]. The complexity of the microvascular environment of tumor is clearly shown by the blood volume maps (see Fig. 1, 2): for some patients, the outlined ROIs are very large, with areas up to 500.0 mm^2^, as demonstrated by the histogram in Fig. 3.

Nevertheless, this variability allowed us to identify among the perfusion maps those having the highest prognostic power. Using the ROC curves, it was possible to establish the predictive value of each parameter that resulted statistically significant: ***PS***, ***Pat*_*Rsq *_**and ***T*_*peak*_**. Both ***Pat*_*Rsq *_**and ***PS ***were confirmed to be equally reliable metrics for discriminating between malignant and normal tissues, with AUCs of 0.82 and 0.81 respectively, and p_z _value of 0.02. Instead, ***T*_*peak *_**was not found to be significant, with an AUC of 0.68 and p_z _value of 0.11.

The strong relation between ***PS ***and ***Pat*_*Rsq *_**has also been confirmed by the Spearman correlation coefficient (Table 6) and the scatter plot in Fig. 5.

The perfusion studies, both with CT and or MRI, considered by recent studies, can be used for preoperative grading of the gliomas, in particularly for the differential diagnosis of low and high-grade astrocitomas because these technique can provide complementary information about tumor hemodynamics, not available with conventional CT or MR. The potential role of these techniques in follow-up analysis, lies in the differential diagnosis between radiation necrosis and recurrence in patients who have undergone radiotherapy and in the evaluation of the response to the anti-angiogenetic therapy, and its ability to detect the biological effects to treatment by depicting early microvascularization modifications, related to a reduction in microvessel density, before tumor dimension modifications [21-24].

## Conclusion

Tumors are characterized by higher values of all the perfusion parameters. Using statistical analyses both the *PS *and *Pat*_*Rsq *_resulted significant for discriminating between malignant and normal tissue, with comparable prognostic power.

Additional studies, including a greater quantity of data, to differentiate between the patients with high and low grade tumors, or those with radionecrosis and recurrence are warranted.

## Competing interests

The authors declare that they have no competing interests.

## Authors' contributions

AMDN and MC conceived of the study and partecipated in its design and coordination. AV carried out the perfusion CT exams. SM performed the statistical analyses and partecipated in the draft of the manuscript. AF, AP and CMC contributed with the enrollement of patients; in particular CMC enrolled those patients undergoing a surgery or stereotactic biopsy. AM partecipated in the design of the study and selected those patients eligible for a radiotherapy treatment. All authors read and approved the final draft.

## References

[B1] Boone JM (2007). Radiological interpretation 2020: Toward quantitative image assessment. Med Phys.

[B2] Roberts HC, Roberts TPL, Lee TY, Dillon WP (2002). Dynamic, Contrast-Enhanced CT of human brain tumors: quantitative assessment of blood volume, blood flow, and microvascular permeability: Report of two cases. AJNR.

[B3] Di Nallo AM, Crecco M, Ortenzia O, Ordonez R, Abate A, Benassi M (2007). The breast dynamic contrast enhanced MRI: Preliminary results of a quantitative analysis. J Exp Clin Cancer Res.

[B4] Miles KA, Griffiths MR (2003). Perfusion CT: a worthwhile enhancement?. Br J Radiol.

[B5] Hoeffner EG, Case I, Jain R, Gujar SK, Shah GV, Deveikis JP, Carlos RP, Thompson BG, Harrigan MR, Mukherji SK (2004). Cerebral Perfusion CT: Technique and Clinical applications. Radiology.

[B6] Eastwood JD, Provenzale JM (2003). Cerebral blood flow, blood volume and vascular permeability of cerebral glioma assessed with dynamic CT perfusion imaging. Neuroradiology.

[B7] Ding B, Ling HW, Chen KM, Jiang H, Zhu YB (2006). Comparison of cerebral blood volume and permeability in preoperative grading of intracranial glioma using CT perfusion imaging. Neuroradiology.

[B8] Jain R, Ellika SK, Scarpace L, Schultz LR, Rock JP, Gutierrez J, Patel J, Ewing SC, Mikkelsen T (2008). Quantitative Estimation of Permeability Surface-Area Product in Astroglial Brain Tumors Using Perfusion CT and Correlation with Histopathologic Grade. AJNR.

[B9] Cenic A, Nabavi DG, Craen RA, Gelb AW, Lee TY (2000). A CT Method to Measure Hemodynamics in Brain Tumors: Validation and Application of Cerebral Blood Flow Maps. AJNR.

[B10] Brix G, Bahner ML, Hoffmann U, Horvath A, Schreiber W (1999). Regional Blood Flow, Capillary Permeability, and Compartmental Volumes: Measurement with Dynamic CT – Initial Experience. Radiology.

[B11] Sahani DV, Kalva SP, Hamberg LM, Hahn PF, Willett CG, Saini S, Mueller PR, Lee T (2005). Assessing Tumor Perfusion and Treatment Response in Rectal Cancer with Multisection CT: Initial Observations. Radiology.

[B12] Molen AJ Van Der, Veldkamp WJH, Geleijns J (2007). 16-slice CT: achievable effective doses of common protocols in comparison with recent CT dose surveys. British Journal of Radiology.

[B13] Axel L (1980). Cerebral blood flow determination by rapid-sequence computed tomography. Radiology.

[B14] Patlak CS, Blasberg RG (1985). Graphical evaluation of blood-to-brain transfer constants from multiple-time uptake data. Generalizations. J Cereb Blood Flow Metab.

[B15] Metz CE (1989). Some practical issues of experimental design and data analysis in radiological ROC studies. Invest Radiol.

[B16] Van Erkel AR, Pattynaia PM (1998). Receiver operating characteristic (ROC) analysis: Basic principles and applications in radiology. Eur J Radiol.

[B17] Sanelli PC, Nicola G, Tsiouris AJ, Ougorets I, Knight C, Frommer B, Veronelli S, Zimmerman RD (2007). Reproducibility of Postprocessing of Quantitative CT Perfusional Maps. Neuroradiology.

[B18] Miles KA (2003). Perfusion CT for the assessment of tumor vascularity: which protocol?. Br J Radiol.

[B19] Newbold K, Castellano I, Charles-Edwards E, Mears D, Sohaib A, Leach M, Rhys-Evans P, Clarke P, Fisher C, Harrington K, Nutting C (2008). An exploratory study into the role of dynamic contrast-enhanced magnetic resonance imaging or perfusion computed tomography for detection of intratumoral hypoxia in head-and-neck cancer. Int J Radiation Oncology Biol Phys.

[B20] Bisdas S, Nguyen SA, Anand SK, Glavina G, Day T, Rumboldt Z (2007). Outcome prediction after surgery and chemoradiation of squamous cell carcinoma in the oral cavity, oropharynx, and hypopharinx: use of baseline perfusion CT microcirculatory parameters vs. tumor volume. Int J Radiation Oncology Biol Phys.

[B21] Schmainda KM, Rand SD, Joseph AM, Lund R, Ward BD, Pathak AP, Ulmer JL, Baddrudoja MA, Krouwer HGJ (2004). Characterization of a First-Pass Gradient-Echo Spin-Echo Method to Predict Barin Tumor Grade and Angiogenesis. AJNR.

[B22] Sugahara T, Korogi Y, Tomiguchi S, Shigematsu Y, Ikushima I, Kira T, Liang L, Ushio Y, Takahashi M (2000). Posttherapeutic Intraaxial Brain Tumor: The Value of Perfusion-sensitive Contrast-enhanced MR Iamging for Differentiating Tumor Recurrence from Nonneoplastic Contrast-enhancing Tissue. AJNR.

[B23] Li KL, Zhu XP, Checkley DR, Tessier JJL, Hillier VF, Waterton JC, Jackson A (2003). Simultaneous mapping of blood volume and endothelial permeability surface area product in gliomas using iterative analysis of first-pass dynamic contrast enhanced MRI data. The British Journal and Radiology.

[B24] Zima A, Carlos R, Gandhi D, Case I, Teknos T, Mukherji SK (2007). Can Pretreatment CT Perfusion Predict Response of Advanced Squamous Cell Carcinoma of the Upper Aerodigestive Tract Treated with Induction Chemotherapy?. AJNR.

